# Integration of B-to-B trade network models of structural evolution and monetary flows reproducing all major empirical laws

**DOI:** 10.1038/s41598-024-54719-0

**Published:** 2024-02-26

**Authors:** Jun’ichi Ozaki, Eduardo Viegas, Hideki Takayasu, Misako Takayasu

**Affiliations:** 1 Department of Computer Science, School of Computing, Tokyo Institute of Technology, 4259, Nagatsuta-cho, Midori-ku, Yokohama, 226-8503 Japan; 2 Centre for Complexity Science and Department of Mathematics, Imperial College, London, SW7 2AZ UK; 3 Sony Computer Science Laboratories, Inc., 3-14-13, Higashigotanda, Shinagawa-ku, Tokyo, 141-0022 Japan

**Keywords:** Complex networks, Statistical physics, thermodynamics and nonlinear dynamics, Complex networks, Statistical physics, thermodynamics and nonlinear dynamics

## Abstract

We develop a single two-layered model framework that captures and replicates both the statistical properties of the network as well as those of the intrinsic quantities of the agents. Our model framework consists of two distinct yet connected elements that were previously only studied in isolation, namely methods related to temporal network structures and those associated with money transport flows. Within this context, the network structure emerges from the first layer and its topological structure is transferred to the second layer associated with the money transactions. In this manner, we can explain how the micro-level dynamics of the agents within the network lead to the exogenous manifestation of the aggregated system statistical data en-wrapping the very same agents within the system. This is done by capturing the essential dynamics of collective motion in complex networks that enable the simultaneous emergence of tent-shaped distributions in growth rates within the agents, together with the emergence of scaling properties within the network in the study. We can validate the model framework and dynamics by applying these to the context of the real-world inter-firm trading network of firms in Japan and comparing the results of the statistical distributions at both network and agent levels in a temporal manner. In particular, we compare our results to the fundamental quantities supporting the seven empirical laws observed in data: the degree distribution, the mean degree growth rate over time, the age distribution of the firms, the preferential attachment, the sales distribution in steady states, their growth rates, their scaling relations generated by the model. We find these results to be nearly identical to the real-world data. The framework has the potential to be transformed into a forecasting tool to support decision-makers on financial and prudential policies.

## Introduction

Over recent years, social, economic, transport, and logistics networks have been enthusiastically investigated. Distinct models under the context of complex networks were proposed in order to explain the underlying mechanisms associated with the interaction among agents within these systems. We deemed these studies to be essential for both theoretical and practical uses, as it can be argued that network science provides a stable and consistent generic framework to analyse the complex emerging phenomena observed in these networks. Here we give some examples: detecting the rumour diffusion on the SNS network^1,2^, systemic risk in the banking network under economic crisis^3,4^, and the disorder in supply chains^5,6^. All these works unveiled significant practical and theoretical issues that require careful consideration.

Specifically within the economic and strategic management context, early research^7,8^ on large inter-firm trade networks adopting network and graph theory methods provided significant contribution to the field by bringing to spotlight specific elements of complex economic systems that were clearly understood but not articulated in depth. Indeed, a recent article^9^ by researchers within the field elegantly contextualise the importance of the analysis of supply chain networks for policy making, and it further argues for the development of large datasets on a global scale.

Here, we highlight the research and work carried out by academics in conjunction with the National Bank of Belgium^10^. Based on records from VAT sales, an extensive dataset was produced on the inter-firm trade network within Belgium in order to derive classic input and output matrices and to analyse the length of supply chains within Belgium. The results provide particular insight into how over 80% of the Belgium are fundamentally dependent on production export, even though they only operate directly at local market level.

Another example is the seminal research from the New England Complex Systems Institute^11^ on Corporate Competition^12^. In this cited study, the authors make use of traditional methodologies associated with complex networks^13^ to evaluate the dynamics of a data set on competition networks among firms in the United States. More specifically, the authors compare the results of two micro-dynamics competition models to key properties of the real-world, encompassing companies across various sectors and industries in the United States. This is done in order to highlight three specific mechanisms of network formation, namely (1) a geographical-mechanism, (2) the effects of size of companies in shaping the links (i.e. the traditional preferential attachment mechanism) and (3) interdependence between firm-size dynamics and the competition network formation, which is essentially modeled as a Gibrat-like stochastic process, through a feedback mechanism.

Whereas significant elements of the research described above, such as the role of the preferential attachment mechanism, and the interdependence between firm-size dynamics and network structure, our research has significant differences in focus leading to very distinct model dynamics, and yet complimentary results. To start, we are less preoccupied with the geographical influence on network formation. This is not to say that these are not relevant, as the impact of geography within Japan is indeed part of a separate research from the authors. However, they have much reduced role and distinct impact when compared to much larger countries such as the United States for reasons of density, area and shape. Firstly, in relation to density, over 40% of the total number of Japanese transactions involve companies located in Tokyo prefecture. As importantly, the large majority of the other companies are located in few prefectures such Osaka, Nagoya and Kyoto that have excellent fast connections through the rail infrastructure. Furthermore, as a mountainous country, densities in Japan are concentrated within the coast. The transport infrastructure, combined the with the geographical nature of the country leads to distortions to analysis based solely on Euclidean distances. Secondly, Japan is 26 times smaller than the US, indeed it is smaller than the State of California alone which makes most cities within easy reach by air transport (i.e. within one hour). Instead of geography, we are much more preoccupied with the effects of mergers and acquisitions that, together with mortality, have a very significant impact of both trade and competition networks^14,15^, and it is not a focus within the referenced study. In addition, our model is based on the principle that the sales growth is a function of the structural changes in the network in line with established research^7,8,16–19^ within the field, which is distinct from the approach adopted by the research on Corporate Competition^12^.

In addition to the distinct emphasis above, our data is based on a physical and monetary exchange network among one million companies, from the very large to the smallest firm, and therefore very distinct from a much smaller non-physical competition network. The distinction is particularly noticeable on the balance between in and out degrees as well as the power law like behaviour of the various quantities within the system that is not observed with the competition network. As behaviours differ, the specific methods associated with growth mechanism are also distinct.

Shifting focus from economics to the complex systems context, fundamental models for generating networks with specific properties have been studied at length. Barabási and Albert^13^ succeeded in realising scale-free networks, in which the degree distribution follows a power law based on the concept of preferential attachment (which resembles the early works on cumulative advantage process^20,21^), where the probability for a node to get a link is proportional to the degree of the node. Miura et al.^22^ observed the significant influence of the preferential attachment mechanism in a real-world empirical data set (i.e. the inter-firm trading network of firms) in Japan. However, the latter also shows that preferential attachment in isolation fails to explain the negative 1.3 power law exponent observed for the cumulative degree distribution of the Japanese inter-firm trading network, as the Barabási-Albert model^13^ inevitably leads to a constant and negative exponent of 2. Moreover, it was also noted the merging (or coagulation) of nodes (representing agents) is an intricate process within several dynamic complex systems that go beyond the simple dynamics of creation and destruction of nodes. Therefore, an enhanced model of the mergers and acquisitions (i.e. coagulation) of companies was proposed. A subsequent study^23^ provides further insights in relation to the network structure (i.e. in view of 3-body motif distributions).

However, while those previous studies succeeded in replicating the static properties of existing networks, they had little focus on the their related dynamical or temporal properties. As importantly, these studies were predominantly focused on the interactions of the agents^13,22,23^, and in very few cases^16,17,24^ on the consistency between the statistical properties of the network and the statistical properties of intrinsic quantities of the agents. In any event, they did not attempt to explain how the micro-level dynamics of the agents (or nodes) within the network are connected to the exogenous manifestation of the aggregated market (or system) statistical data en-wrapping the very same agents within that market (or system).

Therefore, it is within this context that we place the motivation for our study: We are primarily preoccupied with two elements: (a) going a step beyond a point-in-time network analysis to enhance the understanding of the essential mechanisms associated with the evolution of the statistical properties of these networks, and (b) to couple into a single two-layered model framework the dynamics of interactions between agents (i.e. the temporal network structure layer) to the intrinsic quantities characterising the agents (i.e. money transport layer). To this extent, our work is to be seen as a new framework that uses a number of the building blocks of modelling methodologies previously proposed. However, the framework wraps these into a congruous and coordinated framework by extending, refining and fine-tuning a number of the underlying processes. In this manner, we can explain both the temporal statistical properties of the network as well as those of the agents within a single modelling foundation.

Here, it is worth emphasising that our research is not a simple aggregation of models. The modifications to the above methods may look at times subtle in nature (as we attempt to minimise unnecessary changes), but these are fundamental to (a) have better alignment to the real-world dynamics and (b) provide results that are fully consistent with all core emerging properties of the system.

Our study is structured as follows. Firstly, within the “Methods” section, we introduce the conceptual framework underpinning our evolving complexity model, explain the process flow, and detail the mechanics associated with the distinct layers. The model parameters are derived through a step-by-step iterative process based on the real-data analysis and also by ensuring that the fundamental quantities associated with the empirical four laws for the temporal network structure and the empirical three laws for the network money transport are always maintained in a consistent manner. Secondly, “Results” section is divided into the outputs related to the temporal network structure layer and the network money transport layer. Both sections are subdivided by an analysis of the key quantities characterising the actual data set, their corresponding fitting elements and the distinct model simulation results. We finish with a “Discussion” section where we further explore our key findings and potential industrial usage of the modelling framework.

## Methods

### Structure and combination flow

**Figure 1:**
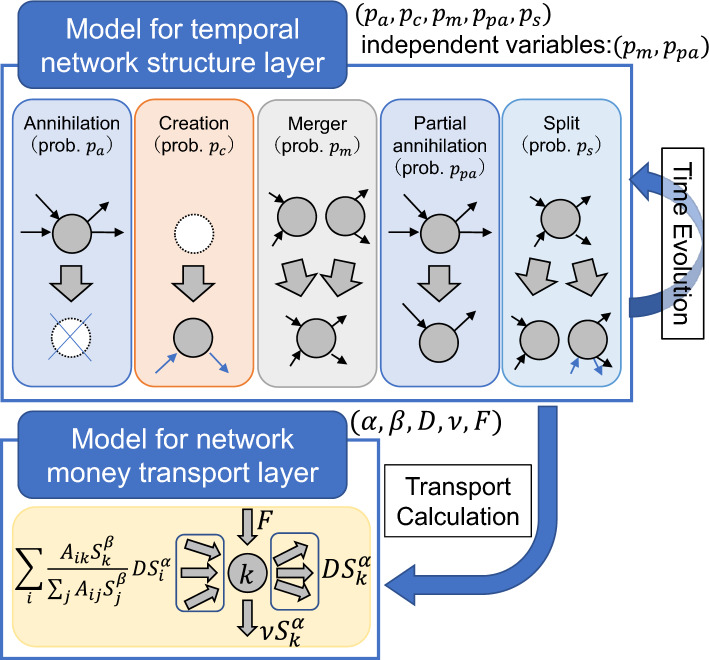
The overview of the methodological framework. A single framework unifies two distinct yet connected layers: (1) the Model for temporal network structure layer and (2) the Model for money transport layer. The model parameters are indicated on the right side of each box (layer).

**Figure 2:**
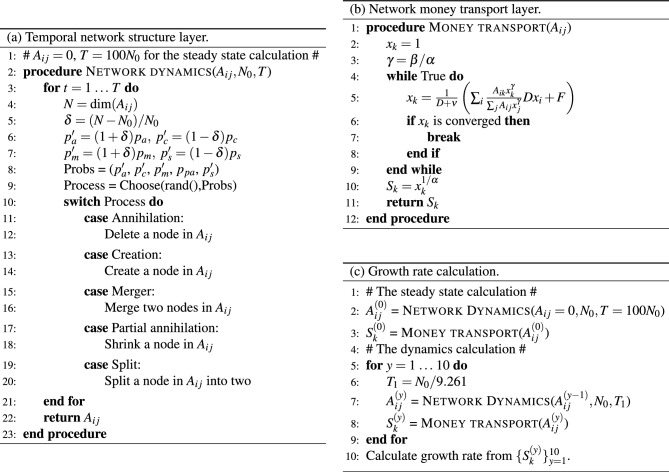
An overview of the framework through the modelling pseudocodes. Item (a) on the left side consist of the pseudocode for the temporal network structure layer. $A_{ij}$ represents the network adjacency matrix of the inter-firm trade network with $N_0$ as the target node number and *T* being total number of calculation time steps. We start with a generating at steady state, and we prepare the $N_0$ nodes initially, setting $A_{ij} = 0$ and $T=100 N_0$. The network dynamics procedure starts with the node number *N* corresponding to the dimension of $A_{ij}$. As *N* varies, the node annihilation/creation bias is changed by multiplying $\delta$ by the corresponding parameters. The simulation ends after *T* time steps. Item (b) on the top right side corresponds to the pseudocode for the network money transport layer. The resulting network adjacency matrix $A_{ij}$ from (a) is an input within the layer. After a number of iterative loops, a solution for $x_k = S_k^\alpha$ is reached, and the sales of each company $S_k$ corresponds to the final output. Item (c) at the bottom right is the pseudocode that calculates the yearly growth rate of the firms for 10 years. The network is initiated by a steady state of the temporal network model. The time evolution is simulated through the temporal network model, and the sales $S_{k}^{(y)}$ on each year *y* are estimated using the money transport model each year.

**Figure 3:**
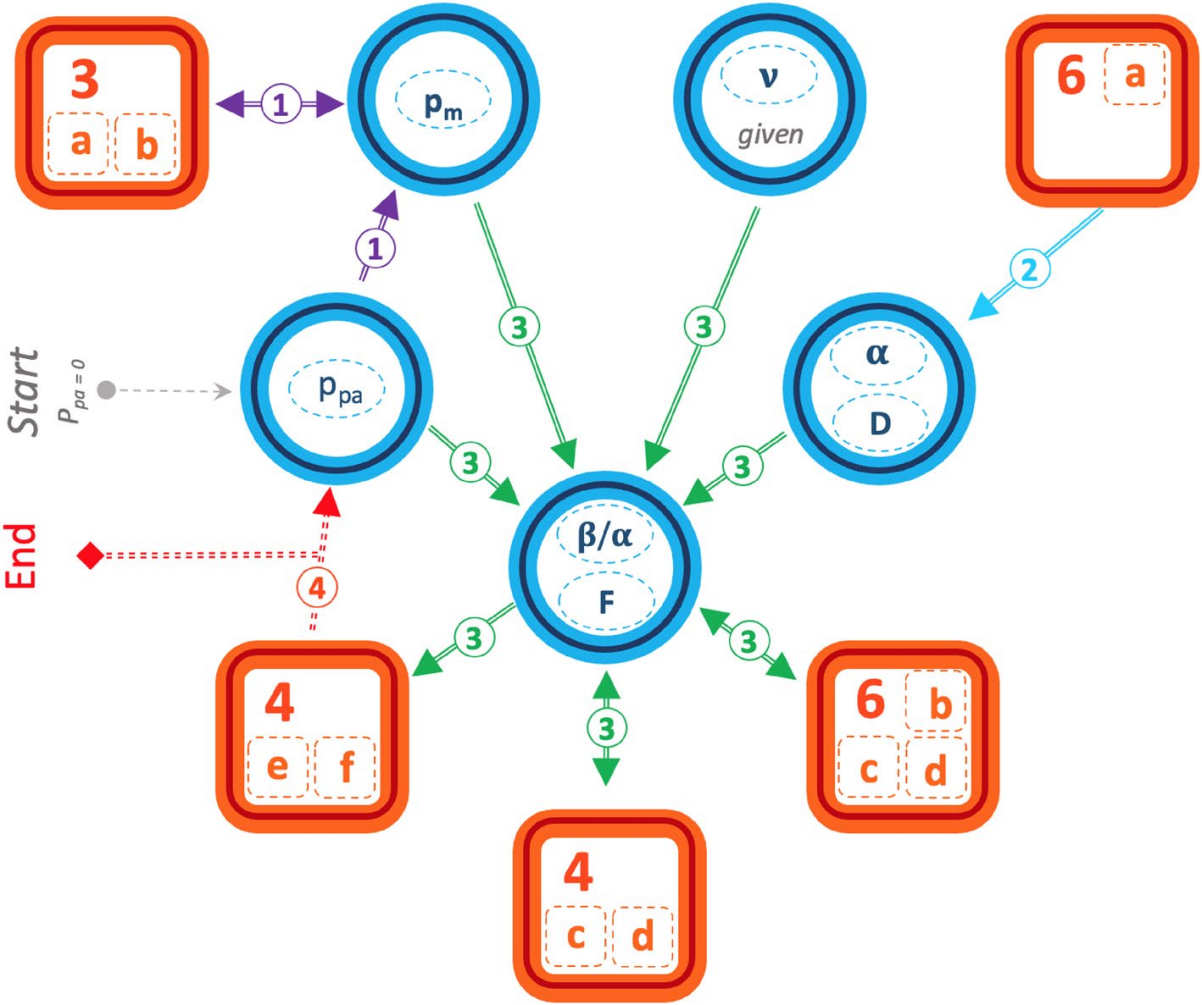
Parameters setting process flow. Each of the parameters is represented by the oval shapes within blue circles, where those within the same circle are calculated together at the same time. The rectangular orange shapes characterise underlying data, with the corresponding figures referenced within. The compound lines represent the processes that lead to the generation of each parameter. Of these lines, dual arrow lines correspond to a fitting process based on the comparison between simulated and actual data, whereas single arrows represent parameters simply derived from actual data or already-determined parameters. The dotted lines correspond to decision-making process points. The flow starts with the setting of $p_{pa} = 0$. (1) corresponds to seeking the optimal value of $p_m$ by comparing the actual data to the simulation results, calculated solely within the temporal network structure layer. (2) represents the actual data analysis where $\alpha$ and *D* from previous studies are validated, with $\nu$ given. (3) corresponds to the computation of the fitting parameters $\beta$ and *F* based on the comparison between the actual data (dual arrows) and simulated results from the best-fitted feeding parameters. The process generates the simulated values for the tent-shaped growth curves (rectangle 4e,f). These values are then compared to the actual data in (4). The process ends if the results are aligned. If not, $p_{pa}$ is changed upwards (or downwards in case of over-estimation) and the whole process restarts.

**Figure 4:**
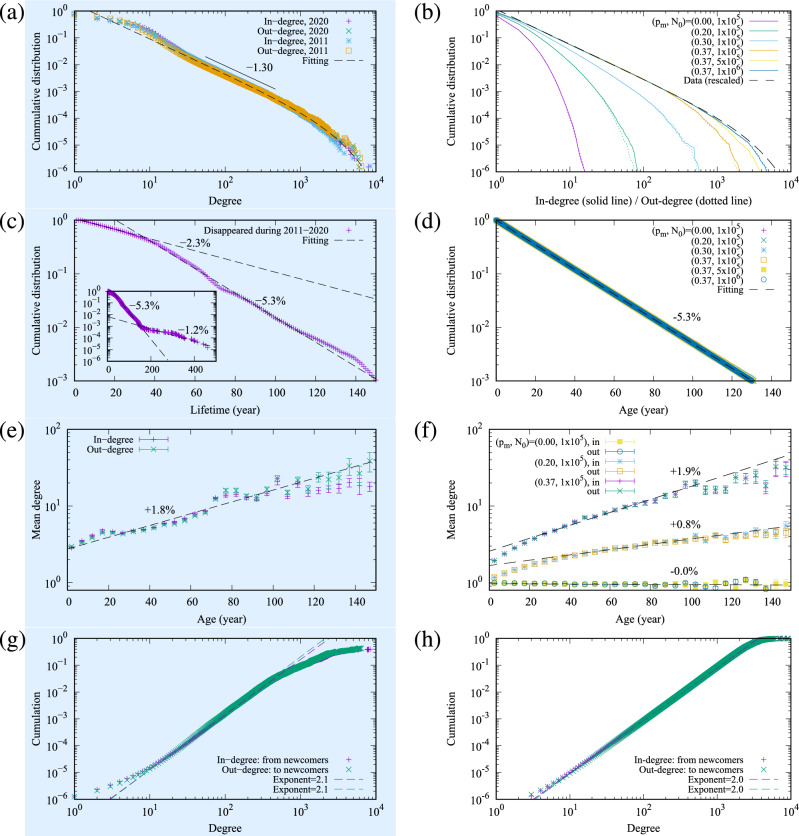
Empirical four laws for the temporal network structure layer. The light-blue background (left side) relates to plots for key quantities characterising the actual data set and corresponding fitting elements, whereas the right side corresponds to the simulation results, compared to the actual data. Figures (**a**) and (**b**) correspond to the in-degree and out-degree cumulative distribution functions (CDFs). Figure (**a**) shows the empirical inter-firm trading network in Japan in 2011 and 2020, where the exponent $-1.30$ can be observed. A fitting function $C e^{-a k} k^{-b}$ for 2020, with parameters $(a,b,C)=(3.80\times 10^{-4},1.30,1.83)$ is also plotted. Figure (**b**) shows the results of simulated networks of 10 samples at $p_{pa}=0.02$. A data fitting result, where *C* is adjusted to $C=1.15$, is also plotted as “Data (rescaled)”. Figures (**c**) and (**d**) correspond to the lifetime distribution of firms in Japan. The lifetime in Fig. (**c**) is estimated by the earliest of the foundation or establishment date, to the bankruptcy or closing date of a company as observed between 2011 and 2020. The rates 2.3%, 5.3%, and 1.2% are according to the disappearance rates in the time span [0,30], [40,140], and $[140,\infty )$, respectively. Figure (**d**) relates to the results of simulated networks of 10 samples with $p_{pa}=0.02$. The decay constant is consistent with the data. Figures (**e**) and (**f**) represent the mean degree, plotted as a function of the firm age. Figure (**e**) is the real data for Japan in 2020, showing an average increase of 1.8% per year. The dotted line corresponds to the fit of an exponential function in [0, 100]. Figure (**f**) shows the simulation results for different $p_m$. They are fitted in [0, 100] at $p_m=0$ and [40, 100] at the other parameters. The average degree growth rate is an increasing function of $p_m$. Figures (**g**) and (**h**) show the preferential attachment of the newcomers. Figure (**g**) is the real data of the cumulation of the preferential attachment probability for Japan in 2020, with an exponent of 2 (we fit the data in [10,200]), thus indicating that the probability of a newcomer attaching to a firm is proportional to that firm’s degree. Figure (h) plots the simulation results which are consistent with the empirical data.

**Figure 5:**
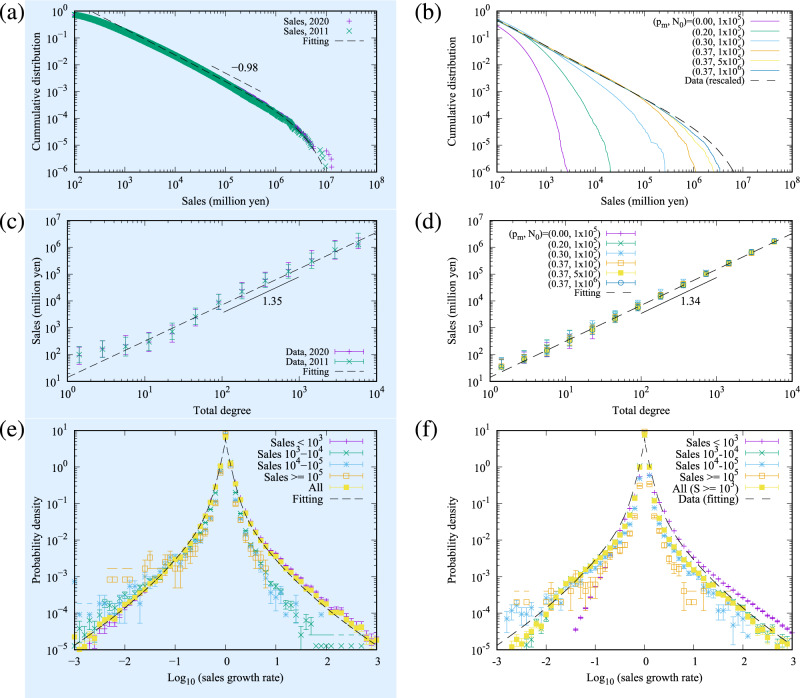
Empirical three laws for the network money transport layer. The light-blue background (left side) relates to plots for key quantities characterising the actual data set and corresponding fitting elements, whereas the right side corresponds to the simulation results, compared to the actual data. Figures (**a**) and (**b**) correspond to the cumulative distribution function (CDFs) of the firm sales *S*. Figure (**a**) shows the empirical data in Japan in 2011 and 2020, where the exponent $-0.98$ can be observed. A fitting function $C e^{-a k} k^{-b}$ for 2020, with parameters $(a,b,C)=(3.39\times 10^{-7},0.98,188)$ is also plotted. Figure (**b**) shows the results of simulated networks of 10 samples at $p_{pa}=0.02$. A data fitting result, where *C* is adjusted to $C=130$, is also plotted as “Data (rescaled)”. Figures (**c**) and (**d**) correspond to the scaling relation between degree and sales. Figure (**c**) is a plot of the median for the empirical inter-firm trading network in Japan in 2011 and 2020. The bars show the quantiles, with the dotted line representing a power-law scaling. Figure (**d**) relates to the results of simulated networks of 10 samples at $p_{pa}=0.02$. Figures (**e**) and (**f**) represent the probability density of the log growth rate of firm sales for different sales scale levels. Figure (**e**) corresponds to the empirical data in Japan during 2011-2020. The fitting function is $2.93e^{-6.91|x|^{0.525}}+3.63 e^{-15.4|x|}$, where *x* indicates the log growth rate. We plot the cases of $S<10^3$, $10^3\le S < 10^4$, $10^4\le S < 10^5$, $10^5 \le S$, and all *S*, where *S* represent the total sales. It is observed that firms with large sales have a narrow distribution. Figure (**f**) represents the simulation results at different scale levels with $(N_0,p_m,p_{pa})=(10^6,0.37,0.02)$. The fitting function of (**e**) is also plotted for reference. We show the cases of $S<10^3$, $10^3\le S < 10^4$, $10^4\le S < 10^5$, $10^5 \le S$, and $10^3\le S$ as “All ($S\ge 10^3$)”. We discard the firms of $S<10^3$ in “All ($S\ge 10^3$)” because the model has the limitation of not calculating the smallest firm sales.

This study is structured as one single framework that unifies and generates full consistency between two distinct yet connected layers based on modelling methodologies from previous studies: (1) the Model for temporal network structure layer and (2) the Model for money transport layer. The schematic figure shown in Fig. 1 provides a graphic summary of the structure with its embedded process flow and variables to be used. Also, the model pseudocodes are shown in Fig. 2. In addition, Fig. 3 provides a zoomed-in and sequential schematic representation of the process flow for the parameters setting, where the order of derivation and dependencies are explained by connecting each of the parameters to the Figs. 4, 5 and 7 within the “Results” section.

In terms of general process flow, the Model for temporal network structure layer dynamically generates a simulated network that evolves in time. Within this layer, it is possible to calculate the key properties concerning the network structure, such as degree distributions and the age of nodes. By deriving the parameters from the real network data and inputting these into the model, we are able to generate a synthetic network that effectively replicates the actual network properties.

Outputs from the synthetic network are transferred to the Model for the money transport layer in order to estimate the total annual sales (referred to simply as ‘sales’ thereafter) of every node (or firm). Again, by deriving the parameters from the real-world sales distribution data, we are able to simulate the total sales as well as the time-dependent growth rates.

This is done through an iterative process where the initial parameters are then slightly adjusted (to reduce differences between the model results and the real data), and the process is re-initiated until convergence is achieved.

With regards to the parameters setting process, as detailed in Fig. 3, the flow starts with the setting of $p_{pa} = 0$. Within the temporal network structure layer, an optimal value of $p_m$ is then obtained by comparing the actual data to the simulation results (Fig. 4a,b). $\alpha$ and *D* from previous studies are validated based on the actual data analysis (Fig. 7a), with $\nu$ given. It follows that parameters $\beta$ and *F* are fitted based on the comparison between the actual data (Figs. 5c,d and 7b–d) and simulated results from the best-fitted feeding parameters. The simulated values for the tent-shaped growth curves (Fig. 5e,f) are generated and compared to the actual data. The process ends if the results are aligned. If not, $p_{pa}$ is changed upwards, and the whole process restarts in an iterative manner until convergence is achieved (2 times in our case). An example of the parameter fitting loop is given in the Supplementary Note 1.

Importantly, through the two-layer approach, these simulations provide results that are not only consistent with the real financial data distribution observations but also with the underlying embedded network structure. To our knowledge, this approach is fundamentally a novel method for the analysis of companies’ performance and network structure.

### Data sources: financial sales and transaction level data

All data was sourced from the Corporate Credit Report (CCR) database, provided by Teikoku Databank, Ltd. (TDB)^25^, one of the largest corporate credit analysis companies in Japan. The data set contains financial (annual sales, number of employees, borrowings, etc.) and corporate information (foundation/establishment dates, shareholding, etc.) as well as business-to-business trade level data for almost the entire set of Japanese firms. Our study relates to data from 2011 to 2020.

The trade level data is used to construct empirical inter-firm trading networks (the benchmark for the outputs for temporal network structure layer and related parameterisation). The data has financial records and corporate information for 604,759 firms (or nodes) in 2011 and 653,659 in 2020. The total number of recorded business-to-business trades (or edges/links) was 3,683,716 in 2011 and 4,192,092 in 2020. Correspondingly, the real network mean degrees are 6.09 and 6.41, respectively. As a representation of the network, the money flow defines the link direction, where a firm *i* buying (outflow) a product from a supplying firm *j* (inflow), is represented by the link $i\rightarrow j$, and the network representation as adjacency matrix is set to $A_{ij}=1$.

Moreover, the financial annual sales data of the firms (or nodes) is used to compute the distribution of total sales and associated growth rates (the benchmark for the outputs for the money transport layer and related parameterisation).

### Model for temporal network structure layer (A)

We propose a fundamentally new model for the temporal network structure layer, derived from the model originally developed by Miura et al.^22^. We maintained the general structure of the network with regard to the nature of the nodes (representing firms) and edges (representing money flows), as well as the stochastic processes within. However, the model dynamics, which were originally based on three core underlying processes (i.e. (a) annihilation, (b) creation and (c) merger of nodes), is expanded and enhanced, being complemented by two important additional processes: (d) the partial annihilation of nodes, and (e) the split of nodes. This is done for a better reflection of the more complex real-world dynamics of business and the observations from the data.

We explain: Firstly, it is a fact that some companies simply disappear (in particular, the smallest ones) in the event of bankruptcy or closure. However, it is also true that a significant number of these companies may also continue to trade, albeit normally in much-reduced size, with limited business activities and a still significant number of counterparties. One example to illustrate this case is that of Lehman Brothers International Europe Ltd.^26^. The company is part of one of the largest bankruptcies in history. Although the bankruptcy occurred in 2008, the company continues to exist today (i.e. 2023). The number of counterparties, however, reduced from an estimated 22,000–10,000 within a few months after the event, and it is fewer than 100 as of today. Indeed, within the US, there are even distinct legal bankruptcy routes: In ‘Chapter 7’, a company is liquidated, and the assets are sold to pay creditors. In contrast, in ‘Chapter 11’ a bankrupt company is restructured (but not liquidated) under the supervision of court-appointed trustees. Therefore, the introduction of partial annihilation aims to address the existing realities of ‘Chapter 11’ cases, which does not result in a total disappearance of a company. Secondly, it is also widespread for companies to split. This may be for the reasons of a public offering of a business (the co-called IPOs) or simply due to regulation, general restructuring or partners that simply fell out with each other. A high-profile example is that of Lloyds TSB Bank. In 2013, Lloyds and TSB were split into two separate banks, following a European Commission ruling requiring the entity to divest part of its business on (lack of) competition grounds^27^. In short, the two additional processes are a reflection of real-world dynamics that are important and relevant, but that were not present in the previous models.

In summary, our model dynamics consist of five kinds of elementary stochastic processes: (a) annihilation, (b) creation, (c) merger, (d) partial annihilation, and (e) split. The respective probabilities, $p_a$, $p_c$, $p_m$, $p_{pa}$, and $p_s$, represent a chance for the process to occur and a node (firm) to be selected under certain rules as follows.

Within the annihilation process (a), a node is uniformly and randomly selected to disappear. All existing links attached to the selected node are then deleted.

The creation process (b) generates a new node *i* within the system. Two exiting nodes $j_{1}$ and $j_{2}$ are then selected under the preferential attachment mechanism to form one out-link $i\rightarrow j_{1}$ and one in-link $j_{2}\rightarrow i$. Thus, the attachment probability for selecting node *j* is $\frac{k_{j}+1}{\sum _{z=1}^{N} (k_{z}+1)}$, where $k_j$ is the total degree of the node *j*, and *N* represents the set of all existing nodes within the system.

The merger process (c) consists of the uniform and random selection of a node *i* and the selection of another node *j* under the same preferential attachment mechanism defined within the creation process (previous paragraph). Once both nodes are selected, all links related to *i* are transferred to *j*, and node *i* is eliminated.

The partial annihilation process (d) also uniformly and randomly selects a node within the system which continues to exist. However, it partially loses some of its links. The ratio of the lost links is assumed to obey the uniform distribution in the range [0,1]. The links chosen for deletion are also uniformly and randomly selected.

The split process (e) is essentially structured as a reversal of the merger process, where a node, chosen under the preferential attachment mechanism, transfers a proportion of its links to a newly created node. The proportion of links to be transferred is structured to approximate the reversal mechanics of the merger process. Here, a merged node comprises a node of degree $k_1$ picked up by the preferential attachment $p_1(k_1) \propto (k_1 + 1)p_0(k_1)$ and a node of degree $k_0$ chosen at uniformly and randomly $p_0(k_0)$, where $p_0(\cdot )$ is the degree distribution, and $p_1(\cdot )$ is of the preferential attachment. The degree of the merged node is $k=k_1+k_0$, under a simplifying assumption of no overlaps between the links during the merger process. To mirror a reversal of the merger process, the split process needs to divide the node into portions $k_1/(k_1+k_0)$ and $k_0/(k_1+k_0)$ in order to restore the two nodes following the same probability. Considering the property of the preferential attachment, we can assume $k_1 \gg k_0$ and $p_1(k_1) \simeq p_1(k)$. This effectively means that choosing the original merger node equates to choosing the split node in this specific case, enabling us to approximate $k_1$ as *k*. Thus, we let the ratio of the given links by the split node be $r = k_0 / (k + k_0)$, where *k* is the degree of the split node and $k_0$ is the degree of the reference node. These are taken uniformly and randomly from the network. The reference node is just selected to determine the ratio, and it is not affected by the process itself.

Our core simulation maintains *N* (the set of all existing nodes within the system) steady at a relatively constant quantity. This essentially means that the total probability of creation of nodes $q_{c}$ must be equal to the total probability of destruction of nodes $q_{d}$. Therefore, the parameters ($p_a$, $p_c$, $p_m$, $p_{pa}$, $p_s$) need to be constrained and be mathematically interrelated in order for *N* to be kept at around a target node number $N_0$. It follows that

1 $$\begin{aligned} p_a + p_m = p_c + p_s, \end{aligned}$$

where the left side of the equation relates to the individual processes involving the destruction of nodes $q_{d}$ and the right side concerns those related to the creation $q_{c}$, noting that $p_{pa}$ is the only process that does not involve any change in the number of nodes.

As at every time step a process must occur, the following condition also exists

2 $$\begin{aligned} p_a+p_{pa}+p_c+p_m+p_s = 1. \end{aligned}$$

Replacing the elements of Eq. (1), and making use of *q* = $q_{c}$ = $q_{d}$, we obtain

3 $$\begin{aligned} q = \frac{1-p_{pa}}{2}. \end{aligned}$$

Essentially, this means that without any further adjustment, at every time step, there is a probability *q* that the total number of nodes *N* will either increase or decrease. Therefore, *N* would be subject to fluctuations obeying Brownian motion and will vary in time, resulting in a system beyond a steady state.

In order to maintain stability, we are therefore required to slightly modify the probabilities within the actual simulation process

4 $$\begin{aligned} p_a^\prime =(1+\delta )p_a \ , \ p_c^\prime =(1-\delta )p_c \ , \ p_m^\prime =(1+\delta )p_m \ , \ p_s^\prime =(1-\delta )p_s, \end{aligned}$$

where $\delta =\frac{N-N_0}{N_0}$ corresponds to the node imbalance.

Also, as the mechanisms of mergers and splits are essentially the reverse of one another, we also assume these to be symmetric, $p_m = p_s$. This approach reduces the parameters solely to $p_m$ and $p_{pa}$ as all others can be derived from Eqs. (1) and (3) above.

**Figure 6:**
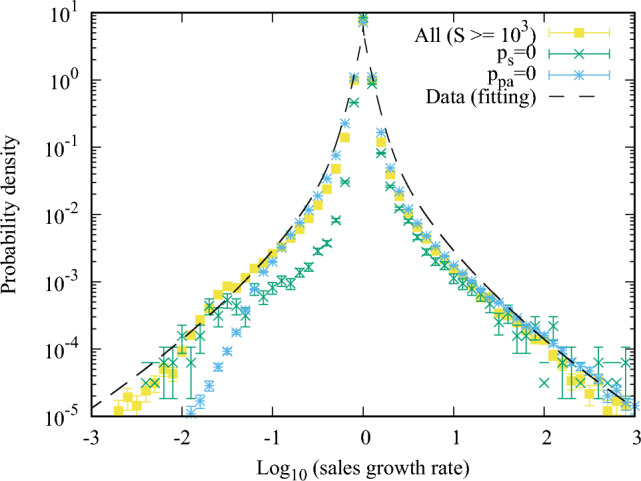
The probability density of the log growth rate of firm sales for different cases of parameters. The plot shows the case of $10^3\le S$ in the simulation as “All” as in Fig. 5f as well as the fitting function of Fig. 5e for reference. The non-symmetric cases of merger and split, $(p_m,p_s,p_{pa})=(0.37,0,0.02)$ is also plotted as green lines and marks. It can be observed that the negative side shrinks and the symmetry breaks. The case where partial annihilation is suppressed, $(p_m,p_s,p_{pa})=(0.37,0.37,0)$ is shown by the light blue lines and marks. The density in the negative-side tail is smaller than in the partial annihilation case ($p_{pa}=0.02$).

**Figure 7:**
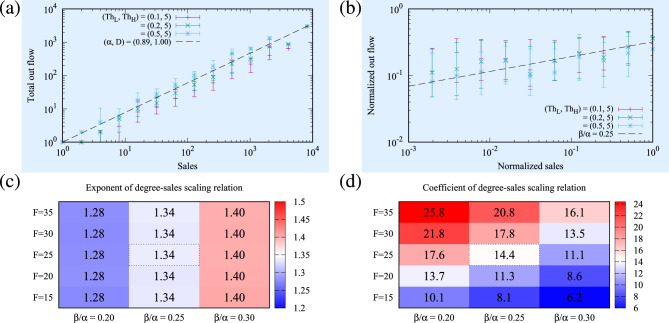
Illustration for the parameter estimations within the network money transport layer. Subplot (**a**) shows the sensitivity and best fit for the parameters $\alpha$ and *D*, where axis y represents the median of the total outflow of money from the bucket of nodes *i* as a function of the sales $S_i$ of the bucket of nodes *i* in 2011–2020 in Japan. In order to reflect the sensitivity of the range selection for solely preserving consistent data, we show the bars for three distinct data ranges, [0.1,5], [0.2,5], and [0.5,5]. The parameter set ($\alpha = 0.89$, $D = 1$) is justified within the thresholds arbitrariness. Subplot (**b**) indicates the reasonable estimation range for parameter $\beta /\alpha$ for the range [0.2,5]. We consider node *i* and related outflows, plotting the normalised sales of the counterparty *j*, $s_j^{(i)}=S_j/\sum _k A_{ik}S_k$ and normalised outflow $\varphi _{ij}=f_{ij}/\sum _k f_{ik}$, where $f_{ij}$ is a flow amount from node *i* to node *j*. The dotted line is estimated when $\beta /\alpha = 0.25$. Subplot (**c**) corresponds to a heat map for different levels of parameters *F* and $\beta /\alpha$ where higher levels of colours (either red or blue) indicate the error of the exponent *a* in the scaling relation $S=A k^{a}$ between the degree *k* and sales *S* (when comparing the model result to the empirical value 1.35, as plotted in Fig. 5c,d). The numbers within each box correspond to the resulting exponent *a* obtained for the modelling combinations of *F* and $\beta /\alpha$. Ten sample networks are generated using the parameter set $(N_0,p_m,p_{pa})=(10^6,0.37,0.02)$ to calculate the scaling relations. Subplot (**d**) is a similar heat map to (**c**) where the error of the coefficient *A* in the scaling relation $S=A k^{a}$ between the degree *k* and sales *S* is shown instead. The empirical value of *A* is 14.2.

The parameter derivation for the temporal network structure layer begins with setting the probability of the merger process $p_m$ to be consistent with the real degree distribution (Fig. 4a,b), whilst maintaining the probability of partial annihilation $p_{pa}$ equal to zero. Parameters for the money transport layer (as described below) are then derived. An iterative process introduces increasing values for the probability for the partial annihilation process $p_{pa}$ (and therefore adjusting the other network parameters according to Eqs. (1)–(3)) to best fit the negative tail of the log growth rate of real sales (Fig. 5e,f). The process is fully repeated (2 times in our case) until convergence is achieved. To avoid complexity and redundancy in visualising the parameter estimation, we only plot the final fitting results in all relevant figures within this paper (Figs. 4, 5, 6, 7). For information, the results from the first iteration are largely aligned to the results for “$p_{pa}=0$” as shown by the blue points in Fig. 6.

We have also analysed the following special cases: (a) Moore’s model^28^ where $p_m=p_{pa}=p_s=0$, and (b) Miura’s original model^22^ where $p_{pa}=p_s=0$. In addition, we will consider the cases where either only the split or the partial annihilation processes are included (i.e. where $p_{pa}=0$ and $p_{s}=0$, respectively) in order to show the need for both processes to be implemented to reliably replicate the actual data, as further explained in the “Results” section.

In all cases, we generated networks that converged to a steady state. We started with $N_0$ nodes ($10^6$ in the final simulation) with no links and calculated the model time step for $T_{{{\text{st}}}} = 100N_{0}$ times, which updated the node state 100 times on average, leading the network to a steady state^22^.

In Fig. 2a, we provide the pseudocode for the model for the temporal network structure layer. The initial condition is $N_0$ nodes without any link for the steady state of the network, whereas we start with a given network for the calculation of the firm growth from a time point. One sample of the simulation consists of the predetermined number of time steps, with each of them looking through the following five processes: (a) annihilation, (b) creation, (c) merger, (d) partial annihilation, and (e) split. At each step in the calculation loop, one process is chosen at the probabilities $p^\prime _a$, $p^\prime _c$, $p^\prime _m$, $p_{pa}$, and $p^\prime _s$, respectively. In each process, the corresponding dynamics are applied to the system, as previously explained.

### Model for network money transport layer (B)

With regards to the money transport layer, the mathematical method from the original proposition suggested by Tamura et al.^18^ is kept unchanged as no fundamental modifications were deemed necessary. However, a partial reparameterisation is carried out in order to ensure consistency with the full set of data available. This means that the original parameters were either validated or modified to reflect our larger and richer data set.

Variations of the gravity model have been used in economics and social sciences, for instance, in the context of estimations of trading amounts between countries^29,30^. This study adopts the expanded gravity model for the money flow between firms suggested by Tamura et al.^18^ since it best fits the existing data set and our objective to build the total sales (an intrinsic attribute of a node) from ‘b-to-b’ relationships (edge attributes).

We also emphasise that, to achieve our goals, we place significant emphasis on replicating the tent-shaped distributions of the growth rate in sales given that distributions resembling this shape format are widely observed in complex systems^31,32^. Specifically, within economic systems, Stanley et al. and other researchers^31^ indicated that the growth rate distribution of the firms is tent-shaped, close to symmetry in each scale, and the typical width of the distribution is scaled by the firms’ total sales. Furthermore, the tent-shaped distribution results from a random multiplicative process of independent components in the firms; the generalised central limit shows the stable distribution of the growth rate, which is close to symmetric tent-shaped^32^. It follows, therefore, that any model attempting to explain the evolving features of an inter-firm trading network must be consistent with these observations as well as with the scaling relations observed both at network interaction and agent levels.

As already described, we use the money transport model based on the gravity interaction model^18,19,33^ for the sales estimation. The money flow defines the link direction, where a firm *i* buying (outflow) a product from a supplying firm *j* (inflow) is represented by the link $i\rightarrow j$, and the network representation as adjacency matrix is set to $A_{ij}=1$. The original gravity interaction model^18,19,33^ approximates that the total money flows out of a firm is proportional to the firm sales to the power of $\alpha \sim 0.9$. It also indicates that the quota of the money flows to each trading partner is proportional to the partner’s sales to the power of $\beta \sim 0.3$. The inflow from and the outflow to the environment (i.e. not captured by the network) are represented by *F* and $\nu S_k^\alpha$, respectively. In that case, the simultaneous equations of the balance of the money flow at a stationary state are given as^18^

5 $$\begin{aligned} \sum _i \frac{A_{ik} S_k^\beta }{\sum _j A_{ij} S_j^\beta } D S_i^\alpha - (D+\nu )S_k^\alpha + F = 0, \end{aligned}$$

where $A_{ij}$ is the adjacency matrix of the trading network, $S_k$ is the total sales of the firm *k*, $D S_k^\alpha$ is the total flow out of the firm *k*, $\beta$ is the exponent of the weight of each outflow, $\nu$ is the strength of the flow to the environment, and *F* is the flow from the environment to each node. The model determines the sales of all firms once the network and the parameters $(\alpha , \beta , D, \nu , F)$ are given. The annual money flow from node *i* to node *k* is given as

6 $$\begin{aligned} f_{ik} = \frac{A_{ik} S_k^\beta }{\sum _j A_{ij} S_j^\beta } D S_i^\alpha . \end{aligned}$$

The solution of this non-linear equation (Eq. 5) is calculated by the following repeated substitution until convergence is achieved, which corresponds to the Jacobi method in Linear algebra:

7 $$\begin{aligned} S_k^\alpha \leftarrow \frac{1}{D+\nu } \left( \sum _i \frac{A_{ik} S_k^\beta }{\sum _j A_{ij} S_j^\beta } D S_i^\alpha + F \right) . \end{aligned}$$

The parameter determination begins with estimating $\alpha$ and *D* by analysing the actual data by plotting the total outflow of each firm as a function of its sales. As some level of inconsistency exists in the data (e.g. an apparently not suitable case that the total outflow is 10 times larger than the annual sales), only data within a certain range $[{\text{Th}}_{{\text{L}}} ,{\text{Th}}_{{\text{H}}} ]$ for the ratio of the total outflow to sales were used in the analysis. We adopted three ranges, $[{\text{Th}}_{{\text{L}}} ,{\text{Th}}_{{\text{H}}} ]$ = [0.1,5], [0.2,5], and [0.5,5]. From the plots in Fig. 7a, the best-fit parameters $\alpha$ and *D* slightly vary depending on the range selected, but the parameters set $(\alpha , D)=(0.89,1)$ in the previous study^18^ confirmed to sit comfortably within these range boundaries and therefore kept unmodified.

Once the above parameters are set, we follow on by plotting the normalised money outflows $\varphi _{ij}=f_{ij}/\sum _k f_{ik}$ (where $f_{ij}$ is a flow amount from node *i* to node *j*) as a function of the normalised sales of counterparty *j*, $s_j^{(i)}=S_j/\sum _k A_{ik}S_k$. This is done in order to identify the reasonable boundaries of $\beta$ unperturbed by the sales scale. We observe that $\beta$ is estimated as $\beta /\alpha \simeq 0.25$ as shown in Fig. 7b. We then refine the parameter $\beta$ and define *F* in order to obtain a reasonable fit for the real data. The adequacy of the fitting is shown in Fig. 7c,d, where the exponents and coefficient in the scaling relation between the degree and sales (as plotted in Fig. 5c,d) in each parameter set are visualised and represented by the heat maps of the errors of the exponent and coefficient in the scaling relation. Also, we checked the suitability of $\nu$ from the previous study simultaneously.

The pseudocode for the model for the network money transport layer is shown in Fig. 2b. The input for the model is the transaction network $A_{ij}$ calculated in the temporal network structure layer. For example, the calculation of the sales growth rate requires the sales of each firm in each year. To do this, we prepare the network for each year from the temporal network structure layer, and we simulate the sales using the networks. The simulation is essentially the iterative looping over of simple substitution of the sales vector $x_k = S_k^\alpha$ to the right side in the Eq. (7) to update $x_k$. The vector $x_k$ is finally converged in the case of the parameter sets used in the study.

## Results

The results section is divided into the outputs related to (A) the temporal network structure layer (Fig. 4) and (B) the network money transport layer (Figs. 5 and 6). Both sections are subdivided by

- (left sides of Figs. 4 and 5, light-blue background) an analysis of the key quantities characterising the actual data set with fitting functions, which determines the base parameters ($N=10^6, p_m=0.37, p_{pa}=0.02$, $\alpha =0.89, \beta /\alpha = 0.25, D=1, \nu =0.1, F=25$) from the process described in the *Methods* section; and
- (right sides of Figs. 4 and 5) the distinct model simulation results, compared to the actual data, and the difference between simulations departing from the base parameters above; and
- (Fig. 6, separately) highlights the results of the modelling simulations when parameters related to new processes of partial annihilation and split of nodes are turned-off (i.e. $p_{pa}$ and $p_{s}$ set to zero in isolation).

### The temporal network structure layer (A)

Each horizontal pairs of plots within Fig. 4 correspond to a key property characterising the four empirical laws of a network structure. These are: the degree distributions in Fig. 4a,b, the lifetime/age distributions in Fig. 4c,d, the mean degree growth over time in Fig. 4e,f, and the preferential attachment in Fig. 4g,h.

#### Data analysis (A1)

We start with the degree distributions in Fig. 4a on the top left row as the most fundamental property of a network. It is noted that the cumulative distribution function (CDF) obeys a shape similar to a power law with an exponent of -1.30. Moreover, splitting the data between in-degree and out-degree provides very similar distributions. The fitting function is a truncated power law function of the degree *k*, $F(k)=C e^{-a k} k^{-b}$, where the exponential factor describes a cutoff from the system size. The fitting parameters in Fig. 4a were $(a,b,C)=(3.80\times 10^{-4},1.30,1.83)$.

We follow on by plotting the lifetime distribution of the firms that disappeared in 2011–2020, Fig. 4c. The data is consistent with an exponential distribution with the mean disappearance rate of 2.3% for the short span of 30 years, 5.3% for the longer span of 40–140 years, and 1.2% for the longest over 140 years. The reciprocals of the timescales correspond to the average firm disappearance rate per year. While the timescale for the intermediate span of 40–140 years was in line with previous research by Miura et al.^22^, our work highlights a new feature: the timescale varied with time and changed abruptly around 1880 and 1985. These years are thought to be aligned with periods of major changes in the economic background of Japanese history: the Meiji Restoration was in 1868, and the high yen recession in 1986, which led to the asset price bubble collapse in 1992.

The plot within the third row, Fig. 4e, shows the firm growth in degree over time. We observe an exponential growth on average, with the mean growth rates both in-degree and out-degree around 1.7%, as reported by Miura et al.^22^.

Lastly, within the bottom row, Fig. 4g, we investigate the preferential attachment from the newcomers. Suppose that a newcomer node comes into the system accompanying a few links which connect to existing nodes and that the connection probability $p_i$ to the existing node *i* is conditioned only by the degree $k_i$ as $p_i=p(k_i)$. To investigate the function *p*(*k*), we plot the cumulative $P_+(k)= \sum _{k^{\prime }=0}^{k} p(k^{\prime }) = \sum _{k^{\prime }=0}^{k} N(\text {new}\rightarrow k^{\prime }) / N(k^{\prime })$ estimated by the empirical data in Fig. 4g, where $N(\text {new}\rightarrow k^{\prime })$ is the number of links from the newcomer nodes to the existing nodes of degree $k^{\prime }$, and $N(k^{\prime })$ is the number of nodes of degree $k^{\prime }$. The figure indicates that the preferential attachment was proportional and consistent with the previous study^22^ as the simulation was proportional to the degree to the power of 2.

#### Model simulation (A2)

We plot the degree distribution in Fig. 4b at $p_{pa}=0.02$ and various combinations of ($p_m, N_0$). At $p_m = 0.0$, the model was close to Moore’s model^28^. The degree distribution, as expected, did not obey a power law. As $p_m$ is increased, the tail of the distribution stretches, and the power law-like behaviour emerges. The power-law exponent of $-1.3$, derived from the empirical data, is obtained when $p_m=0.37$. The cutoff of the power law stretches as the node number $N_0$ increases. The degree distribution of the empirical trading network, as well as its power law tail, is largely replicated at the parameter set $(N_0,p_m,p_{pa})=(10^6,0.37,0.02)$. Ten network samples were generated for each parameter set.

Beyond the static property, we move on to the temporal properties of the steady state. The timescale of one step in the simulation was fit by comparing the node disappearance ratio per year between the simulation and the central part of the empirical data (the time span [40,140] in Fig. 4c): $(p_a+p_m)T_{1}/N_0$ equals 0.053, where $T_{1}$ is the number of simulation time steps corresponding to 1 year. It results in the relation $T_{1}=N_0/9.261$ when $(p_m,p_{pa})=(0.37,0.02)$. The reason for choosing the time span [40,140] is that the major firms in the actual network are built up within such a range. The simulated age distribution is shown in Fig. 4d, and the exponential distribution with the same time constant as the actual data over 35 years ago was reproduced.

Within the simulation, we also evaluated the general trend of growth of firms over time. The mean of the in-degree and the out-degree conditioned by the firm age is shown in Fig. 4f. The firm growth rate was apparently dominated by the parameter $p_m$. The difference where the age was close to zero came from the degree of the newcomers in the simulation fixed to 2. Overall, the exponential growth over time of the firm beyond the newcomers was consistent with the actual data at $p_m=0.37$.

Lastly, we confirm the simulation replicates the existence of the preferential attachment mechanism for the newcomers as being proportional to the degree of a connecting firm. Similarly to the empirical data analysis, we plot the cumulative probability for the preferential attachment in Fig. 4h, where the exponent 2 is reproduced.

### The network money transport layer (B)

In a similar manner to the previous subsection (A), each horizontal pair of plots within Fig. 5 corresponds to a key property characterising the three empirical laws of sales and money transport. These are: the sales distributions in Fig. 5a,b, the scaling relations between the degree and sales in Fig. 5c,d, and the sales growth rate in Fig. 5e,f, for the monetary properties.

#### Data analysis (B1)

In Fig. 5a, we observe that the cumulative distribution of sales *S* is approximated by a power law with the exponent $-0.98$. We made use of a fitting function similar to the degree distribution process, Fig. 4a. The parameters were estimated as $(a,b,C)=(3.39\times 10^{-7},0.98,188)$.

The scaling relation between degree and sales is shown in Fig. 5c. Total sales *S* largely scales as a function of the degree *k* as $S\propto k^{1.35}$. These properties were consistent with the previous studies of Japanese inter-firm trading networks^16^.

The final property is the firm growth rate $S_{i,y}/S_{i,y-1}$, which is of a temporal nature, where $S_{i,y}$ is the annual sales of the firm *i* and year *y*. The firm growth rate in sales is plotted in Fig. 5e. The log growth rate distribution was approximately symmetric and tent-shaped. However, such symmetry is not maintained when companies are clustered into subgroups of sales orders of magnitude. The figure shows that significant growth for larger firms is less likely than for smaller firms, a fact already observed in other works^14,31^; the variance of the log growth rate decreases with the sales scale.

#### Model simulation (B2)

We observe that the integrated model reproduces the key properties characterising the three empirical laws of sales and money transport, when using the base parameters described in the first paragraph of the “Results” section.

Firstly, the sales distribution is plotted in Fig. 5b. Similar to the degree distribution, the tail of the distribution stretches as $p_m$ is increased and finally the power law-like behaviour emerges; the cutoff of the power law stretches as the node number $N_0$ increases. The model result at the base parameter is very closely aligned with the empirical sales distribution, with an exponent of approximately $-1$.

The simulated scaling relation between degree and sales is shown in Fig. 5d. Here, changes to the parameters associated with the network layer (i.e. $p_{m}$ and $N_{0}$) are largely insensitive as the parameter $\beta$ affects the exponent, and the scale is dominated by *F*.

We previously stated in “Methods” section that we place significant emphasis on replicating the tent-shaped distributions of the growth rate of the elements that are widely observed in those complex systems^31,32^, and that a core objective of our integrated model is to explain the evolving features of an inter-firm trading network where the tent-like shape naturally emerges from these dynamics. As a result, we place significant importance on the results shown in Fig. 5f. Here, starting with the steady state achieved after $T_{{{\text{st}}}} = 100N_{0}$ time steps from the initial state, we continued to simulate the network dynamics to analyse the dynamical properties. The growth rate distribution was calculated by simulating the time steps corresponding to 10 years, where the procedure for this calculation is shown in the pseudocode in Fig. 2c. The tent-shaped distribution naturally emerged as observed in Fig. 5f. The distributions of all the firms (yellow line) were consistent in both the data and the simulation results. Also, the sales growth rate had a narrower tail on the positive side as the firm sales were more significant. Those two results differed in the positive-side growth rate distribution of the smallest firms. In the simulation, we used the money transport model to estimate the firm sales, which had the limitation of not calculating the small firm sales well because the minimum firm sales in the simulation is determined by $S_k^\alpha \ge \frac{F}{D+\nu } \simeq 23$ from Eq. (5). As a result, the smallest firm category was not simulated correctly. However, the simulation explained the real data well for the firms with $10^3$ sales or over. We also note that the more minor firm sales were more volatile, as our data analysis and the previous studies reported^31^.

Lastly, in order to highlight the importance of the two additional model dynamics proposed by our study within the temporal network structure layer—namely, the partial annihilation of edges and the split of nodes—we show results where the split process is turned off ($p_s=0$) (yellow in Fig. 5f) and separately where partial annihilation is also turned off $(p_{pa}=0)$ (blue in Fig. 5f). Both results show significant worsening in the fitting to the actual data. In particular, where $(p_{pa}=0)$, the tail on the negative side of the growth rate is smaller than the actual data, which demonstrates that the split process in isolation does not fully explain the shrinking of firms. In short, both processes are required. Moreover, if we were to break the symmetry of the merger and split processes ($p_{m}>0,p_{s}=0$), the symmetry of the growth rate is also broken down. This can be observed in Fig. 5f, where the log growth rate in the non-symmetric case, $p_m=0.37$ but $p_s=0$ is plotted. It is possible to note that the distribution on the negative side is shrunk, and it largely becomes not tent-shaped.

## Discussion

We developed an integrated model framework based on two distinct yet connected layers of modelling methodologies that were previously subject to a reasonable amount of research but in isolation from each other.

Through our approach, we were able to create a methodology that is fundamentally and solely dependent on the micro-level dynamics of interactions among agents to replicate the quantities and explain the emerging phenomena arising not only at the structural network level (network properties), but also at the structural economic market level (agent intrinsic properties) in both case from a static as well as temporal perspective. In essence, it could be argued that our work provides a solid example of a comprehensive simulation method of a complex system with a significant level of consistency.

Moreover, we highlight the importance of the micro-level activities of startups, mergers, acquisitions, disinvestment and bankruptcy to the formation of the tent-shaped growth rate distribution of sales of firms that it is commonly observed at the structural economic market level. In particular, the introduction of the split process is indispensable to the formation of the log growth rate symmetry observed in the real data. Here we further detail the scope and analysis within our research. Firstly, this study revisited the preferential attachment mechanism and validated that it is proportional to the node degree as indicated in the previous study by Miura et al.^22^. Hence, we validated the broader existence of the preferential attachment under the Barabási and Albert model. However, an unmodified version of the model would not be consistent and fit the observed degree distribution’s exponent larger than $-3$ (in the cumulative distribution function, $-2$), whereas we can show that the inclusion of the merger mechanism achieves that effect. Here we emphasise again that the inclusion of the merger mechanism is not solely a mathematical solution, it simply aims to reflect the real life dynamics of business where mergers and acquisitions are a constant feature that significantly changes the size and the structure of the supply chain of firms.

Secondly, we also carried out specific additional and target analysis on a small region of Japan, Tokyo’s 23 wards, (see Supplementary note 2), to demonstrate that within our network the geographical distance does not have a significant effect on the degree distribution of firms. These results are distinct from the studies of that of Braha et al.^12^. However, this is not surprising given that (a) the nature of the networks are very distinct (i.e. a real trade network in our study as opposed to a derived competition network that become naturally biased towards larger companies that produce publicly available financial report); (b) the size of the networks are of substantially different scales and (c) the fact that the countries involved (Japan in our case and US in that study) have very significantly different demographics both in terms of area as well as density.

Thirdly, this study has broken the limitations of the previous study by Miura et al.^22^. We investigated the case of no split process and showed that the tent-shaped distribution of the actual growth rate is not achieved without the split process.

As importantly, we also show the limitations arising from potential modelling oversimplifications. This is illustrated by the need to take into account the multifaceted aspects of bankruptcy (i.e. annihilation and partial annihilation of edges) dependent on the sales scale, in order for the tent-shaped growth rate distribution to be closely replicated, including its variance scaled to a degree.

We also believe our parameterisation method, where each parameter to the model is based on a serialised, and iterative, step-by-step process, is essential to a better understanding of the importance, effect, and sensitivity of each underlying dynamical process in isolation. Here, we highlight that it is only by following this process we can identify that the merger process supports the mean growth rate of the system, while the split process makes the log growth rate symmetric. In contrast, if we were to adopt methods aiming at solving all parameters simultaneously through optimisation targets and functions, in the vein of machine learning, the link between each of the individual underlying processes, and their related impact might be missed. We firmly believe that these methods, albeit computationally elegant and trendy at present, tend to be less fit for the purposes of understanding the fundamental system dynamics at compartmentalised, sub-component levels. Though, we accept that this is an area of choice and vigorous debate.

As the framework reliably replicates the key quantities of the complex economic system of firms in Japan, one can speculate that it would be highly feasible to adapt the framework to a forecasting tool based on ‘what-if’ scenarios where the inputs may be essentially driven by financial and prudential policies (government support for companies to avoid full bankruptcy or decision of splitting companies to improve market competition) and the outputs the overall effect to the economy (as growth of sales and total sales as highly associated with GDP growth and value). Some additional level of work and potential refinements would be required, however.

Lastly, we highlight two limitations of our work. Firstly, as described within the “Results” section, the framework does not replicate well the growth rate of very small firms, and further work can be done in the future to refine such shortcomings. Since we observed from a recent, separate work, that geographical trade distance disproportionately affects the behaviour of small firms, we can hypothesise that their sales growth might be highly disturbed. Therefore, future evolution of the proposed framework may include a geospatial perspective. Secondly, it is not possible from the data to directly observe companies that underwent partial bankruptcy and companies that have split. Ideally, we would like to compare the real number of events to those generated by the model (in a similar manner we did for newcomers) so that the parameters could be refined even further. However, this is a difficult limitation to be addressed.

### Supplementary Information

Supplementary Information.

## Data Availability

The data that support the findings of this study are available from Teikoku Databank, Ltd.^25,34^ but restrictions apply to the availability of these data, which were used under license for the current study, and so are not publicly available. Data are however available from the corresponding author M.T. (contact: takayasu.m.aa@m.titech.ac.jp) upon reasonable request and with permission of Teikoku Databank, Ltd.
