# Supplementary material for: Integration of B-to-B trade network models of structural evolution and monetary flows reproducing all major empirical laws
Source: Sci Rep. 2024 Feb 26;14:4628. doi: 10.1038/s41598-024-54719-0 (PMC10897299; doi:10.1038/s41598-024-54719-0)
Supplement: Supplementary file 1 — Supplementary Information. [file 41598_2024_54719_MOESM1_ESM.pdf]

Integration of B-to-B trade network models of structural evolution and monetary flows reproducing all major empirical laws

Supplementary Material

Jun'ichi Ozaki, Eduardo Viegas, Hideki Takayasu, Misako Takayasu

Supplementary note 1 | An example of a parameter fitting loop.

As explained in the main text, the parameter fitting starts with the setting of $p_{pa} = 0$. First, we determine an optimal value of p_m by comparing the actual data to the simulation results. Here, we plot the degree distribution in different p_m in Fig. S1(a). The plot visualises that the optimal p_m is $p_m = 0.37$.

After the parameter p_m is determined, we move on to the parameters β and F (note that $\alpha = 0.89$ and $D = 1$ are validated based on the actual data analysis with $\nu = 0.1$ given, as in the main text). The optimal parameters β and F are estimated through the method given in the main text as $\beta/\alpha = 0.25$ and $F = 25$: the criteria are the difference between the actual data and the simulation in terms of the slope and coefficient in the scaling relation between the degree and the sales.

As a result, the growth rate is calculated in Fig. S1(b). However, in the case of $p_{pa} = 0$, the negative-side tail of the distribution is not realised. Because it is difficult to optimise all the parameters simultaneously here, we determine the best p_{pa} with other parameters given and fixed. We observe that the best parameter is $p_{pa} = 0.02$ from the figure.

However, the change of p_{pa} from $p_{pa} = 0.00$ to $p_{pa} = 0.02$ can affect the other parameters. We have to re-optimize the whole parameter set. Thus, we restart the whole simulation under the new parameter $p_{pa} = 0.02$. We observe that this parameter fixing is enough for converging the entire parameter set: the results at this p_{pa} are shown in the main text. In fact, we confirm that all the properties are consistent between the empirical data and the simulation (see *Results* section in the main text), which justifies the validity of the parameter set.

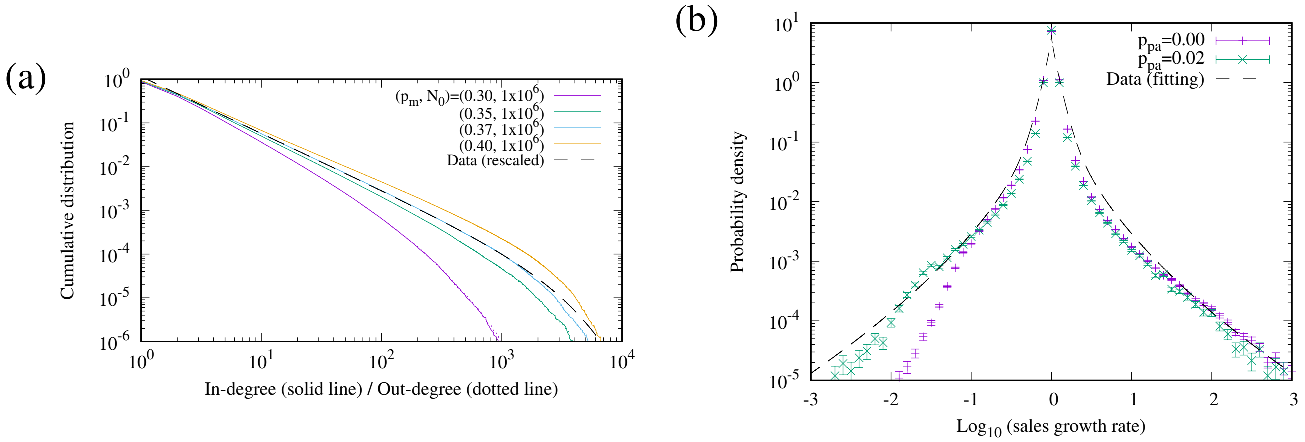

Supplementary Figure S1. (a) The plot shows the results of simulated networks of 10 samples at $p_{pa} = 0.00$. A data fitting result $Ce^{-ak}k^{-b}$ (the parameters are $(a, b, C) = (3.80 \times 10^{-4}, 1.30, 1.15)$) is also plotted as "Data (rescaled)", where only C is adjusted from the actual data fitting. The results of the different cases of p_m are plotted to visualise which parameter best fits the empirical data: $p_m = 0.37$ is the best. (b) The probability density of the log growth rate of firm sales for different cases of parameters. The plot shows the case of $10^3 \leq S$ in the simulation as well as the fitting function of the empirical data for reference as "Data (fitting)". Comparing the simulation results of the cases of $p_{pa} = 0.00$ and $p_{pa} = 0.02$, we observe that the probability of the partial annihilation process causes the negative-side tail in the growth rate.

Supplementary note 2 | Analysis of Tokyo.

We analyse the firm transactions in Tokyo's 23 Wards to compare the result with the case of the whole of Japan. Here, we count only transactions between firms which are both in Tokyo's 23 Wards, and the total degree distributions are plotted in Fig. S2. The plots are similar to each other except for the cutoff region, where the degree is over 10^3. Tokyo's 23 Wards are almost inside a circle whose diameter is 30 km (about 3000km for Japan). In such a small area, the distance is considered unimportant because the cost of transportation is negligible compared to the larger area. Therefore the plot demonstrates that the locational factor does not affect the concentration of the links significantly. Another mechanism, such as a merger, is needed to explain the large exponent of the degree distribution (e.g. the exponent is -2 in the Barabási and Albert model).

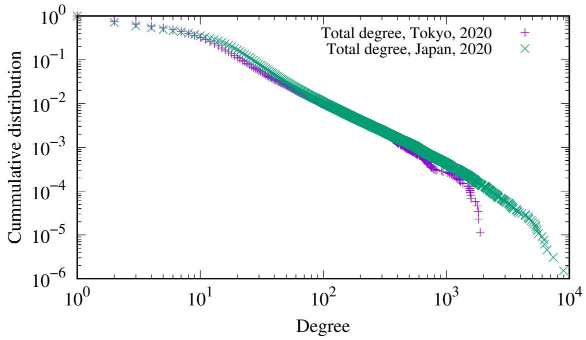

Supplementary Figure S2. The cumulative distribution function of the total degree of the inter-firm transaction networks in the cases of the whole Japan and Tokyo's 23 Wards.
